# Root coverage of a maxillary lateral incisor with gingival recession, gingival stillman’s cleft, bony exostosis, and denture stomatitis: a case report with 3-year follow-up

**DOI:** 10.1186/s12903-022-02068-7

**Published:** 2022-02-11

**Authors:** Chengjie Xie, Yeungyeung Liu, Huimin Yu, Jie Mei

**Affiliations:** Department of Periodontics, Stomatological Hospital, Southern Medical University, 366 JiangNan Avenue South, Guangzhou, 510280 China

**Keywords:** Ginigival recession, Root coverage, Stillman’s cleft, Exostosis, Denture stomatitis

## Abstract

**Background:**

Coronally advanced flap combined with connective tissue graft is considered as the golden standard of root coverage. Although Miller class I recession is considered to get complete root coverage, there are some uncommon conditions in different cases. This case reported a maxillary lateral incisor with a gingival recession, a stillman’s cleft, a bony exostosis and a denture stomatitis.

**Case presentation:**

A 27-year-old female with a gingival recession, a stillman’s cleft and a bony exostosis was treated by coronally advanced flap combined with connective tissue graft technique, and the complete coverage was achieved. Later a denture stomatitis occurred when an acrylic removable partial denture was used, however the gingival margin was not affected. The denture stomatitis disappeared when a new denture with casting palatal plane was produced. In this case of 3-year follow-up, the gingival contour remained stable and the outcome was satisfactory.

**Conclusion:**

Coronally advanced flap combined with connective tissue graft technique is a classic manner to treat gingival recession especially for a long term stability, even when there is a gingival stillman’s cleft, a bony exostosis and a denture stomatitis.

## Background

Gingival recession is defined as the apical migration of the free gingival margin with the consequent exposure of the root surface. The gingival loss may determine esthetic problems, as well as dental hypersensitivity, or non-carious cervical lesions [1]. Moreover the amount of keratinized soft tissue is associated with the inflammatory state of the gingiva and the plaque control [2]. Various surgical techniques have been developed to reach root coverage and to increase keratinized soft tissue. Among them, combined coronally advanced flap (CAF) with connective tissue graft (CTG) has shown the most suitable and predictable results, especially in the long-term follow-up [3]. However, there are some considerations when this technique was applied. As the best as we know, there is very limit literature about the root coverage in gingival recession combined with gingival stillman’s cleft and bony exostosis. This paper reports a case with a gingival recession combined with a gingival stillman’s cleft and a denture stomatitis, in which the details of the surgery process and considerations about the technique were discussed.

## Case presentation

**Fig. 1:**
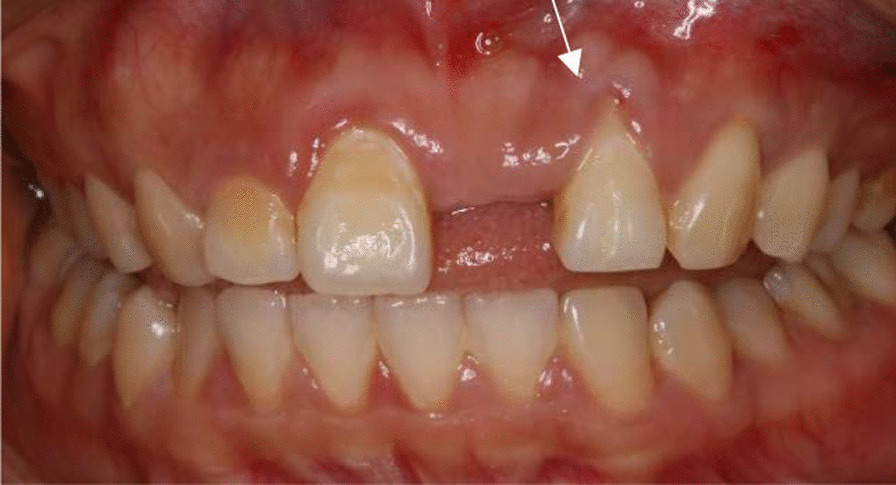
A gingival recession of Miller type I (Cairo RT1) combined with a gingival stillman’s cleft (white arrow) in left maxillary lateral incisor

**Fig. 2:**
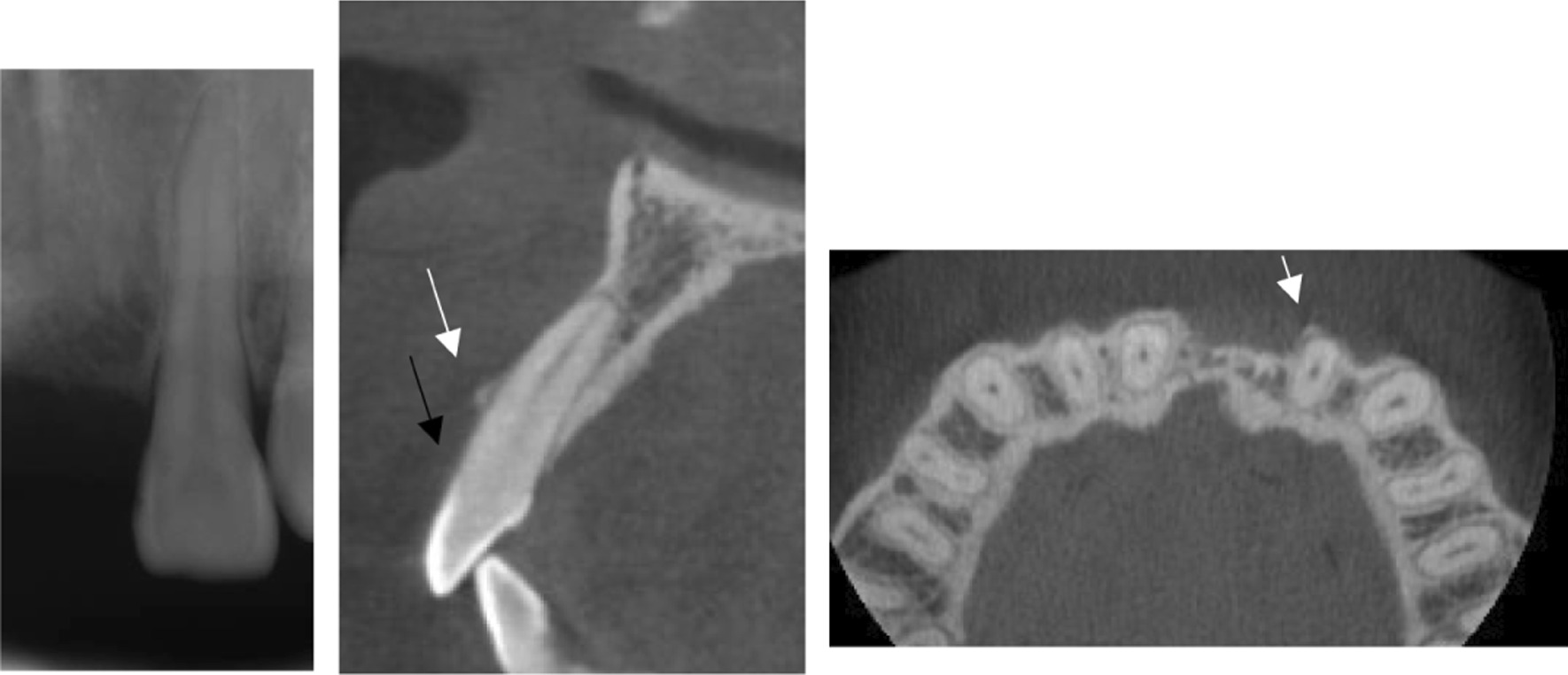
Periapical, sagittal and axial CBCT imaging. **a** No obvious interproximal alveolar bone resorption. **b** Area of bony dehiscence (black arrow) and bony exostosis (white arrow). **c** A bony exostosis in the cross section of cone beam CT (white arrow)

A 27-year-old female, non-smoker, presented with marginal gingival recession and gingival stillman’s cleft of the maxillary left incisor (Fig. 1). Her chief complaint was the lost of left maxillary incisor and she wanted to have a denture. The patient was referred to the periodontal clinic for evaluation and treatment of the mucogingival defect. The lateral incisor presented as type I of Miller’s classification or Cairo RT1 [4] with sufficient keratinized gingival in the apical area. Besides the gingival recession, there was also a stillman’s gingival cleft in the middle point of the gingival margin which could be classified into “complete white gingival cleft” [5]. Moreover, a bony exostosis was noted apical to the gingival cleft. The lateral incisor exhibited proper position in the dentition with no mobility. The periapical radiograph did not suggest any interproximal alveolar bone resorption (Fig. 2). The tooth had a hypersensitivity when checked by cold test. She complained of pain and bleeding when tooth brushing, although she was using bristled toothbrush. She was concerned with the asethetics of a progressive recession defect. She had a removable denture before but she felt uncomfortable and discarded it. She did not present any medical contraindication for periodontal surgery.

The treatment plan was aimed at complete root coverage and soft tissue augmentation. After signing a written consent, a non-surgical periodontal therapy, consisting of oral hygiene instruction, and supra and subgingival scaling was completed. She was also instructed about oral hygiene maintenance at home. According to the consensus [6], she was informed that the tooth had a favorable prognosis due to no significant interdental bone loss.

**Fig. 3:**
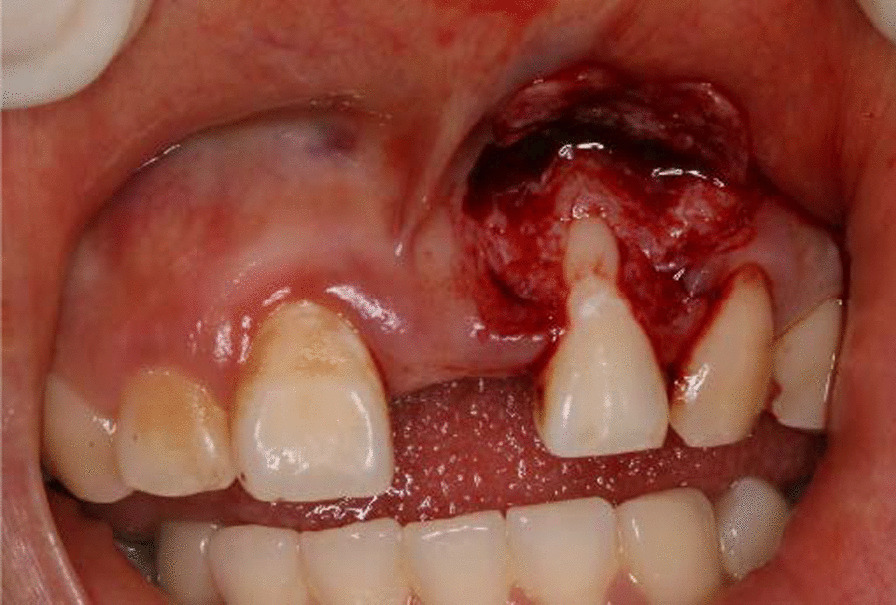
A combination of split-full-split thickness flap was prepared and osteoplasty was performed to reduce the overlying bony exostosis

**Fig. 4:**
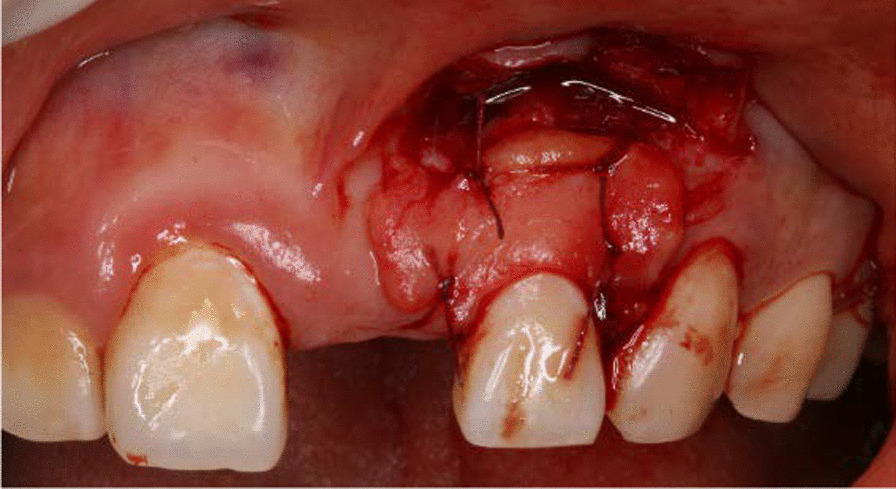
A sub-epithelial connective tissue graft was placed 1–2 mm coronal to the CEJ and stabilized with sutures

**Fig. 5:**
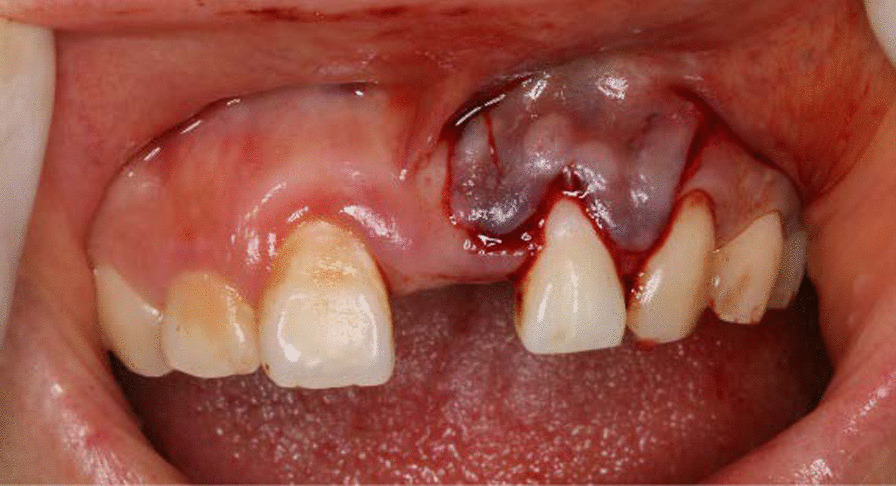
The CTG was covered by a coronally advanced flap

**Fig. 6:**
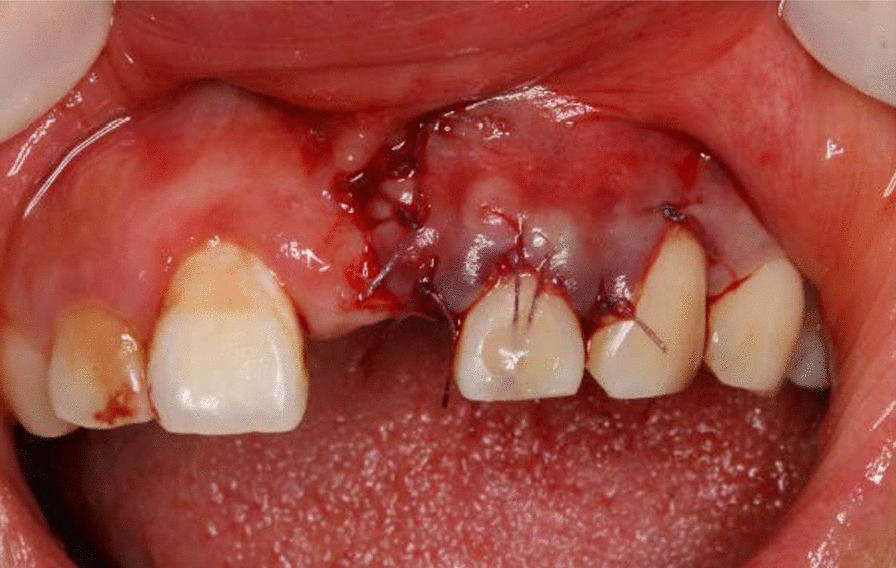
Fixation of the coronally advanced flap

After local anesthesis was obtained, the gingival cleft was completely excised; Then a split-full-split partial thickness flap was prepared to expose the bony exostosis in the apical area and the bony exostosis was reduced away by the hand-piece (Fig. 3), while the root surface was prepared by curette; The CTG was harvested from the keratinized gingiva of the hard palate and trimmed to 1.2–1.5 mm thick; the subepithelial CTG was placed on the root surface coronal to the cementoenamel junction (CEJ) 1–2 mm and stabilized to the periosteum by simple suture with 6-0 suture (Fig. 4); The partial flap was coronally advanced to cover the CTG and fixed with the papilla with 5-0 suture by sling suture (Figs. 5, 6). Antibiotics were administrated to the patient for 5 days, and she was asked to have chlorhexidine rinse for 2 weeks. The sutures were removed after 14 days, and until then she could brush the surgical area in a very gentle rolling method.

**Fig. 7:**
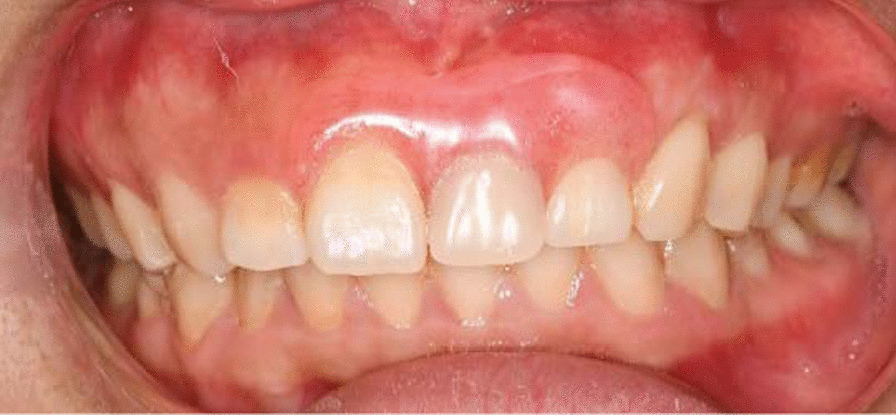
A removable partial denture with acrylic base

**Fig. 8:**
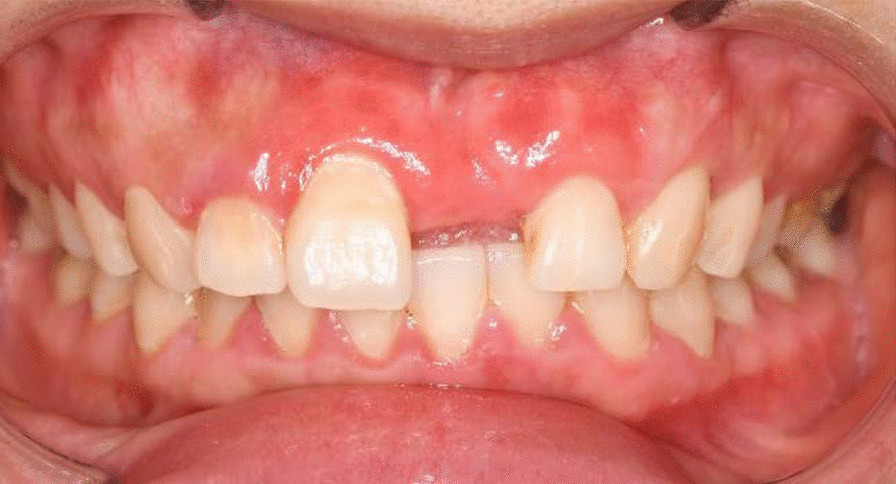
Denture stomatitis resulting from poor oral hygiene and constant use of removable prosthesis

**Fig. 9:**
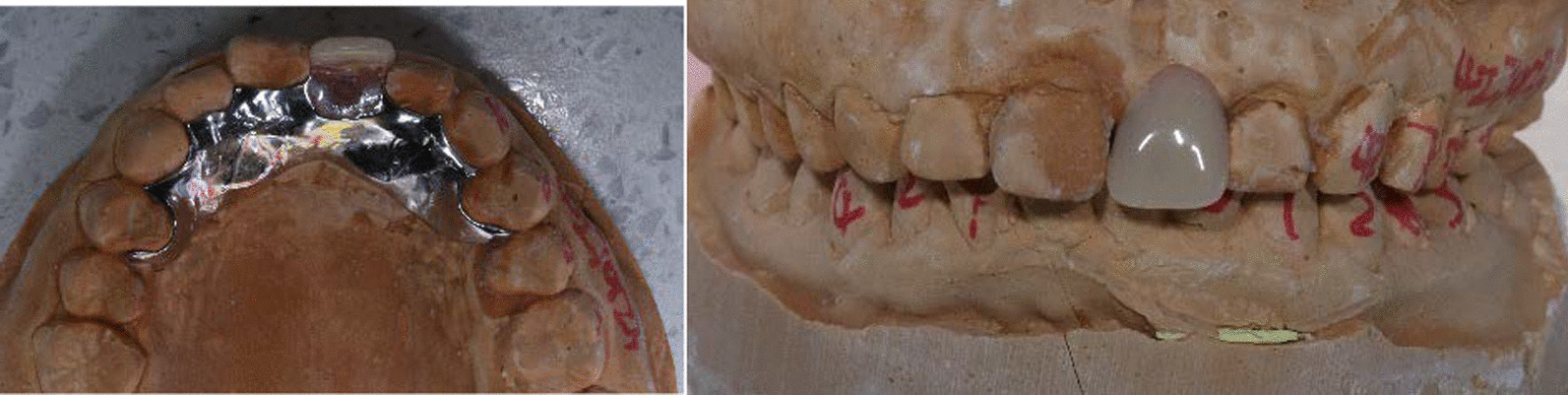
A casting denture without acrylic base around the gingival margin

After 3 month, a removable prosthesis was given to her in the prosthetic clinic (Fig. 7), because the patient didn’t accept a dental implant for financial reasons and surgical fear. When the patient came back at 1 year post-surgery in periodontal clinic, the free gingival margin was still stable as the post-surgery position, with a thicker biotype corresponding to the grafted area. She was satisfied with the gingiva and the aesthetics of the denture, but she complained about the swelling and bleeding around the incisors. Obviously there was a denture stomatitis (Fig. 8). It was presumed that the patient was allergic to the acrylic of the denture, so after a discussion with the prosthodontics, a new denture with a casting palatal plate was re-designed (Fig. 9).

**Fig. 10:**
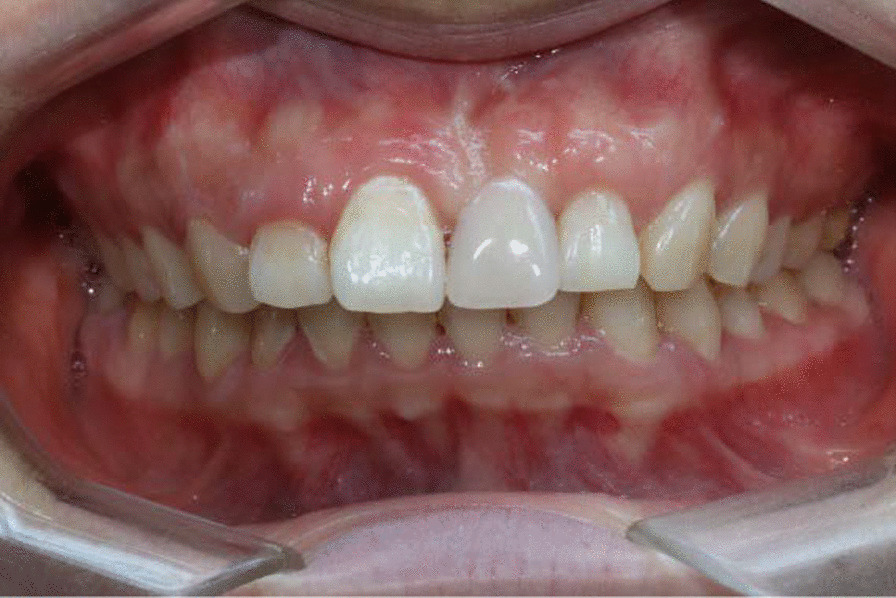
A stable gingival margin and a satisfactory outcome after 3 years

The patient came back again 3 years post-operation. The gingival margin was still stable and the contour was also satisfactory (Fig. 10). The denture stomatitis was resolved, did not recur and the surrounding gingiva presented with shallow probing depths and no bleeding on probing.

## Discussion and conclusions

CAF approach with the addition of a CTG has demonstrated to be the golden standard for root coverage. It could increase the soft tissue thickness augmentation as well as the root coverage maintenance [7, 8]. A long-term stability could be achieved by this technique, and this case with optimal result of 3 years follow-up well supported the conclusion.

According to the prognosis of Miller’s classification, Class I recession could be achieved complete root coverage by means of various technique, especially by CAF + CTG [7, 8]. However when the technique is applied, different conditions may occur, such as non-carious cervical lesions or restorations at cervical area, improper position of the teeth as protrusion or rotation, high frenum attachment and so on. These factors may have aspects on the outcome of root coverage. In this case, the gingival stillman’s cleft, bony exostosis and denture stomatotitis possibly had negative effects on the root coverage. In addition, the oral hygiene was not adequate, but the post- surgical gingival margin kept stable. As a result of the procedure, the modified biotype of the gingiva may have played an important role in preventing further progression of the gingival recession.

Stillman's cleft is a mucogingival triangular-shaped defect on the buccal surface of a root [9]. Hou [5] described cases of stillman’s cleft treated by CAF + CTG which got satisfactory results. Sometimes tunnel technique with CTG is more micro-invasive with no vertical incisions in such esthetic zone [10], however, we applied the partial flap but not tunnel for some special concerns in this case. As a split-full-split partial flap was prepared, the gingival cleft could be excised thoroughly compared with tunnel technique, and the bony exostosis below the gingival cleft could also be thoroughly trimmed, which could not be achieved by other surgical manners.

The patient might be allergic to the acrylic material and she had a denture stomatitis later, furthermore the undesirable oral hygiene might aggravate the stomatitis [11]. The gingivitis disappeared after a new denture was produced. As there was no acrylic material around the gingival margin, it was more suitable to maintain the health of the gingiva.

In this 3-year follow-up, we have noticed a proper tissue augmentation, together with the reestablishment of the natural profile of the affected gingiva. So in conclusion, the classic surgical technique of CAF combined with CTG could harvest a satisfactory and stable outcome for a long time, even when there was an additional gingival cleft and a denture stomatotitis. The biotype improvement of gingva was the critical reason of the long term stability, and the design of prosthesis might also be important.

## Data Availability

The datasets generated during the current study are available from the corresponding author on reasonable request.

## Abbreviations

CAF: Coronally advanced flap
CTG: Connective tissue graft
CEJ: Cementoenamel junction
